# A qualitative assessment of the smoking policies and cessation activities at smaller workplaces

**DOI:** 10.1186/s12889-018-6001-9

**Published:** 2018-09-05

**Authors:** Christine M. Kava, Edith A. Parker, Barbara Baquero, Susan J. Curry, Paul A. Gilbert, Michael Sauder, Daniel K. Sewell

**Affiliations:** 1 Department of Community and Behavioral Health, University of Iowa College of Public Health, 145 N. Riverside Dr., Iowa City, IA 52242 USA; 2 Department of Health Management and Policy, University of Iowa College of Public Health, 145 N. Riverside Dr., Iowa City, IA 52242 USA; 3 Department of Sociology, University of Iowa, 140 Seashore Hall, Iowa City, IA 52242 USA; 4 Department of Biostatistics, University of Iowa College of Public Health, 145 N. Riverside Dr., Iowa City, IA 52242 USA; 5 Health Promotion Research Center, University of Washington, Box 354804, 1107 NE 45th St., Suite 200, Seattle, WA 98105 USA

**Keywords:** Small workplaces, Tobacco control, Smoking, Qualitative, Smoke-free policy, Cessation

## Abstract

**Background:**

To reduce the negative consequences of smoking, workplaces have adopted and implemented anti-smoking initiatives. Compared to large workplaces, less research exists about these initiatives at smaller workplaces, which are more likely to hire low-wage workers with higher rates of smoking. The purpose of this study was to describe and compare the smoking policies and smoking cessation activities at small (20–99 employees) and very small (< 20 employees) workplaces.

**Methods:**

Thirty-two key informants coming from small and very small workplaces in Iowa completed qualitative telephone interviews. Data collection occurred between October 2016 and February 2017. Participants gave descriptions of the anti-smoking initiatives at their workplace. Additional interview topics included questions on enforcement, reasons for adoption, and barriers and facilitators to adoption and implementation. The data were analyzed using counts and content and thematic analysis.

**Results:**

Workplace smoking policies were nearly universal (*n* = 31, 97%), and most workplaces (*n* = 21, 66%) offered activities to help employees quit smoking. Reasons for adoption included the Iowa Smokefree Air Act, to improve employee health, and organizational benefits (e.g., reduced insurance costs). Few challenges existed to adoption and implementation. Commonly cited facilitators included the Iowa Smokefree Air Act, no issues with compliance, and support from others. Compared to small workplaces, very small workplaces offered cessation activities less often and had fewer tobacco policy restrictions.

**Conclusions:**

This study showed well-established tobacco control efforts in small workplaces, but very small workplaces lagged behind. To reduce potential health disparities in smoking, future research and intervention efforts in tobacco control should focus on very small workplaces.

## Background

Considered the leading cause of preventable death, smoking-related illnesses account for over 480,000 deaths each year in the U.S. [1]. The economic costs associated with smoking reach over 300 billion dollars annually [1, 2]. To reduce the negative consequences associated with smoking, many workplaces have implemented smoking policies and smoking cessation activities. Smoking policies are rules and regulations that prohibit smoking [3], while smoking cessation activities are programs designed to increase cessation (e.g., telephone counseling). Both lead to reductions in tobacco use [3, 4].

Compared to larger workplaces, smaller workplaces are less likely to adopt smoking policies and cessation activities. Results from the 2004 National Worksite Health Promotion survey found that only 34% of workplaces with 50–99 employees had a completely smoke-free policy (i.e., smoking completely prohibited on worksite property), compared to 46, 41 and 49% of workplaces employing 100–249, 250–749, and 750 or more employees, respectively. Less than 10% of workplaces with 50–99 employees offered smoking cessation activities, compared to nearly 70% of workplaces with at least 750 employees [5]. A lack of anti-smoking initiatives is especially important to consider at smaller workplaces, many of which report over 50% of their workforce as low-wage [6]. Previous research suggests that people with low socioeconomic status tend to have higher rates of smoking [6], are more likely to start smoking, and less likely to quit [7].

Smaller workplaces may not have these initiatives in place for several reasons. Compared to larger workplaces, smaller workplaces have fewer and less predictable earnings and may lack the resources needed to develop and implement anti-smoking initiatives [8–10]. At the same time, smaller workplaces provide unique opportunities and advantages for tobacco control. Having fewer employees in the workplace may create a “more intimate work culture” [10], which could enhance participation in smoking cessation activities. Since top management is often more accessible at smaller workplaces, support from management for anti-smoking initiatives may be more likely to translate into greater support among employees [10].

### Research gaps

Many of the studies examining anti-smoking initiatives at smaller workplaces are becoming out-of-date. More recent information is needed, given that the landscape for tobacco control has changed considerably over the past 20 years (see Hyland et al. [11]). The prevalence of smoking has been steadily declining from 25% in 1997 [12] to 16% in 2016 [13], with an increasing number of smoke-free laws enacted since then. In 2008, Iowa passed the Smokefree Air Act, which prohibits smoking in most public places, enclosed areas within places of employment, and in company vehicles. Smoking is also prohibited in certain outdoor areas (e.g., grounds of public buildings owned by state government). Areas where smoking is not regulated include: certain hotel rooms designated as smoking, retail tobacco stores, and casino gaming floors [14].

As part of this law, employers are required to post “no smoking” signs or symbols at workplace entrances. Enforcement of the law is governed by the department of public health or its designee. Employers and employees found to be non-compliant face civil penalties (e.g., monetary fine) that increase with the number of offenses [14]. Although it is unclear how many workplaces use these formal procedures when issues of non-compliance arise, previous studies have generally found strong compliance to state smoke-free laws [15, 16].

State and local smoke-free policies can increase smoking ban presence and restrictiveness, as well as alter workplaces norms associated with smoking [15–17]. However, limited research exists on how workplaces have adopted these policies, and the barriers and facilitators experienced when doing so. This is particularly true for workplaces with fewer than 20 employees. Despite the fact that almost 90% of workplaces in the U.S. and 85% of workplaces in Iowa employ less than 20 people [18], a lack of health promotion research persists among this population.

Since evidence shows that the adoption of anti-smoking initiatives increases with workplace size [5], it is important to understand how the context for tobacco control may differ between these and other smaller workplaces. This is especially true for cessation activities, since state law does not require workplaces to offer these activities. Further, since prohibitions on outdoor smoking are not universally required, there may be a need to enact additional policies to fully protect employees at smaller workplaces from the harmful effects of smoking. The specific aim of this study was to describe and compare the smoking policies and smoking cessation activities at small and very small workplaces. Based on previous studies [5, 10, 19, 20], a “small” workplace is defined here as having between 20 and 99 employees and a “very small” workplace as having fewer than 20 employees.

## Methods

### Participants and recruitment

Key informants coming from small and very small workplaces in Iowa participated in qualitative telephone interviews. To be eligible to participate, key informants had to be at least 18 years of age. We recruited key informants via e-mail and telephone through the Business Leadership Network, a university initiative seeking collaborative partnerships with workplaces in Iowa to improve community health [21]. Approximately 730 individuals representing 400 businesses are a part of this Network. Businesses come from a variety of industry sectors, including healthcare, education, manufacturing, and consumer goods. For the purposes of this study, we excluded businesses not classified as small or very small, and individuals whose listed business had no physical location (e.g., board of supervisors). In cases where multiple contacts existed for one business, we contacted the person with the highest ranking or most relevant job title (e.g., CEO, HR Manager). Recruitment occurred between October 2016 and February 2017. Out of the 227 individuals contacted about the study, 32 chose to participate (14% response rate). Fifteen participants worked for a small workplace, and 17 participants worked for a very small workplace. As incentive for participating, key informants were eligible to win one of three $50 gift cards in a random prize drawing.

### Data collection and measurement

The first author (CMK) conducted interviews between November 2016 and February 2017, which were recorded for analysis. Interviews lasted an average of 25 min (SD = 10.87). Interview questions were developed based on topic areas and measures from earlier studies [5, 22–32]. Several items were taken or adapted from the Workplace Health Site Visit Interview [33]. The final interview guide consisted of 28 questions. Participants were asked to provide background information about themselves, their workplace, and their employees. Participants then described the smoking policies and smoking cessation activities existing at their workplace. Participants answered additional questions on the following topics: anti-smoking initiative documentation and promotion; enforcement and compliance; initiative history; cessation activity participation; reasons for adoption or non-adoption; challenges and facilitators to adoption and implementation or barriers and support needed for adoption.

### Data analysis

An independent professional transcriptionist transcribed all interviews. The first author subsequently coded these transcripts in ATLAS.ti [34]. Prior to coding, each transcript was read to gather an initial impression of the data. Analysis followed a directed content approach, which uses existing research to identify key concepts as initial coding categories [35]. After developing initial coding categories, all transcripts were immediately coded. Data that could not be coded with these initial categories were analyzed later, with codes refined and new codes developed during this process.

To increase the trustworthiness of findings, a second coder with qualitative research experience coded a random sample of the transcripts (*n* = 8, 25%). There were no major disagreements in coding between the first and second coder. Minor differences were discussed, and necessary changes were made to the codebook. All transcripts were then coded a second time using the revised codebook. All final codes were examined for patterns and organized into groups to develop themes, an analytic technique common across several qualitative approaches [36]. To help develop themes and facilitate comparisons between small and very small workplaces, site-ordered descriptive matrix displays examining key topic areas by workplace size were created in Excel based on guidelines from Miles and Huberman [37]. Frequency counts for major topics and themes were also calculated.

## Results

### Sample characteristics

Table 1 presents an overview of participant and workplace characteristics. Most participants were female (*n* = 20, 63%) and held top ranking positions within their company, with 11 participants (34%) describing themselves as President, Owner, or Chief Officer. While several participants (*n* = 10, 31%) came from organizations providing health services (e.g., public health agency), participants represented a variety of other workplace industries, including consumer goods, banking, and manufacturing. Twenty-three participants (72%) came from workplaces that offered health insurance and where most employees worked full-time. The estimated mean number of smokers at each workplace was three.

**Table 1:** Participant and workplace characteristics

Characteristic	All workplaces (*N* = 32)	Small workplaces (*n* = 15)	Very small workplaces (*n* = 17)
	n (%)		
Gender			
Male	12 (36%)	5 (33%)	7 (41%)
Female	20 (63%)	10 (67%)	10 (59%)
Job Title^a^			
Human Resources	3 (9%)	3 (20%)	0 (0%)
President, Owner, or Chief Officer	11 (34%)	2 (13%)	9 (53%)
Vice President	3 (9%)	2 (13%)	1 (6%)
Executive Director	5 (16%)	3 (20%)	2 (12%)
Administrator	4 (13%)	2 (13%)	2 (12%)
Prevention specialist	3 (9%)	2 (13%)	1 (6%)
Other	4 (13%)	1 (7%)	3 (18%)
Industry			
Banking	3 (9%)	3 (20%)	0 (0%)
Construction	1 (3%)	1 (7%)	0 (0%)
County services	3 (9%)	2 (13%)	1 (6%)
Health services	10 (31%)	6 (40%)	4 (24%)
Economic or resource development	3 (9%)	0 (0%)	3 (18%)
Manufacturing	2 (6%)	1 (7%)	1 (6%)
Professional association	2 (6%)	1 (7%)	1 (6%)
Consumer goods	4 (13%)	0 (0%)	4 (24%)
Other	4 (13%)	1 (7%)	3 (18%)
Full-time employees			
50% or less full-time	9 (28%)	3 (20%)	6 (35%)
51% or more full-time	23 (72%)	12 (80%)	11 (65%)
Health insurance			
Yes	23 (72%)	15 (100%)	8 (47%)
No	9 (28%)	0 (0%)	9 (53%)
Estimated number of current smokers (mean)	3.48	6.30	1.00

^a^Percentages reach over 100% because one individual held two positions

### Description of anti-smoking initiatives

#### Smoking policies

Of the 32 participants interviewed, 31 (97%) had a formal or informal smoking policy at their workplace (note: since only one participant stated that their workplace had no policy, the results that follow will focus on the 31 workplaces with a policy in place). Twenty-three workplaces (74%) had a policy in writing. All workplaces prohibited smoking indoors; 22 (71%) participants also described outdoor smoking restrictions. Eight participants (26%) described prohibiting smoking in specific contexts (e.g., “no smoking on any calls for service”). The interview guide did not include questions about other forms of tobacco use (e.g., e-cigarettes), but 11 participants (35%) stated that their policy restricted use of these products as well. Fourteen participants (45%) had a smoking policy in place prior to the Iowa Smokefree Air Act.

Most participants described high employee adherence to their workplace smoking policy. Ten participants (32%) indicated that they did not need to enforce the policy, either because no smokers existed within their organization or due to strong compliance. Almost all participants described disciplinary actions that could be taken if employees violated the terms of the smoking policy. Some participants described these disciplinary actions in detail (e.g., “…enforcement of this policy will be addressed through the corrective action policy, which means we have a four-step procedure”). Others described the consequences they believed would occur if an employee violated their smoking policy (e.g., “Probably they’d be written up for the first time”).

#### Smoking cessation activities

Twenty-one (66%) workplaces offered smoking cessation activities to their employees. Activities described by participants included information and referral to other services (12 participants); insurance coverage for cessation medication (8 participants); tobacco services through an employee assistance program or other contracted vendor (7 participants); smoking cessation materials (e.g., magnets, quit kits) (3 participants); smoking cessation classes (3 participants); insurance discounts for non-smokers (1 participant). Fifteen participants (71%) indicated that their workplace had offered activities for at least 5 years. While not all key informants had information on participation, many spoke of very little to no participation by smokers in activities offered (e.g., “Boy I could count ‘em on one hand in the 5 years”).

### Reasons for adoption or non-adoption

When participants were asked to describe the reasons why they had (or had not) adopted anti-smoking initiatives, the following themes arose during thematic analysis: external smoking laws; desire for healthy workplace; organizational benefits; smoking as inappropriate; and lack of initiative need. These themes are described in further detail below, with findings organized by initiative type (smoking policies and cessation activities).

### Smoking policies

Twelve participants (39%) described external smoking laws as a reason for having a smoking policy. In some cases, participants indicated that they did not have a choice to adopt a smoking policy, that these laws required them to restrict smoking in the workplace. Others described adapting or formalizing their smoking policies to remain in compliance with these external restrictions. Six participants (19%) described a desire to have healthy employees and a healthy work environment. Three participants (10%) also spoke about reducing organization costs (e.g., health insurance) associated with smoking.

Six participants (19%) described smoking as inappropriate, with some going on to describe the behavior as being inconsistent with the mission or image of their workplace (e.g., “the image of someone standing at a call for service dealing with…a particular citizen with a cigarette hangin’ out of their mouth or a big chew of tobacco in their mouth, we don’t believe it is professional”). Three participants (10%) spoke about a personal experience or incident related to smoking that influenced their decision to adopt a policy at their workplace:

…my doctor came in, and said he was at one of the other places in town, one of the competitors. And the salesman blew smoke in his face, and he turned around and walked out, and left and came out to us. He told me that story, and I had the no smoking signs ordered the next day...I put ‘em on my main doors, and I just told my help and everyone that, there were no longer any smoking in our business.

#### Smoking cessation activities

Among the 21 participants whose workplace offered smoking cessation activities, seven (33%) described wanting to improve the health of their employees and to support a healthy workforce. Six participants (29%) mentioned some of the benefits for the workplace, including having a more competitive organization, reduced health insurance costs, and increased employee productivity. Three participants (14%) mentioned a desire to help employees quit smoking, recognizing that offering these activities could support cessation (e.g., “If we want people to quit, we have to help them”).

Among the 11 participant workplaces that did not offer smoking cessation activities, six participants (55%) described a lack of need for these activities as the reason for non-adoption, mostly because the workplace had no current smoking employees. Despite not offering activities, five participants (45%) clarified that if employees needed or desired activities, the workplace would be open to offering them. Two participants (18%) described not wanting to force employees to quit smoking, noting that they had been smoking long enough and that offering activities would not help them to quit:

They’re old enough and they know better, and you know what? If they wanna smoke at home, there’s nothin’ I can, I mean, to try to do about it... I’m not gonna waste my time. ‘Cuz they, they’ve both smoked so long, it’s on their own. Like I said, once again, they’re big boys, so, they can do what they want to…You only can beat the horse about so much and that’s it, so.

### Challenges or barriers

Based on participants’ descriptions of the challenges and barriers related to anti-smoking initiative adoption and implementation, the following themes arose: no challenges (lack of initiative need); enforcement and adherence; and lack of employee participation. These themes are described below.

#### Smoking policies

Fifteen participants (48%) described no challenges to adopting and implementing the smoking policy at their workplace, with three reiterating that smoking was simply not an issue. Seven participants (23%) cited issues with adherence to the policy, either among employees or among customers, as a challenge. Two participants (6%) described challenges associated with figuring out what to do with the policy and how to enforce it:

And one is definitely, you know, how do we enforce this? You know, I don’t wanna have to fire or discipline anybody because they’re smokin’ in their car on their lunch break. Nobody wants to do that…But definitely we gotta be able to follow through…I think that’s one of the biggest barriers is people being concerned about how those discipline issues are, or [how] the enforcement part is gonna be taken care of.

#### Smoking cessation activities

Among the 21 participants whose workplace offered smoking cessation activities, nine (43%) indicated that no challenges existed to adoption or implementation. Five participants (24%) described the cost of activities as a challenge or potential challenge. Though not specific to adoption or implementation, four participants (19%) noted difficulties in getting smokers at their workplace to participate in the activities offered:

I think the ones that wants to, that does smoke, they’re gonna wanna smoke so they don’t want stop. Even though I made comments to some of ‘em but. Yeah. I don’t think they’re wantin’ to quit. So. It would be very difficult to get some of ‘em to go through the program.

Among the 11 participants whose workplaces did not offer cessation activities, 10 (91%) stated that no barriers to adopting activities existed, with many of these participants noting a lack of need for activities. One participant indicated that employees who have wanted to quit have been interested in diverse activities, which are more challenging to accommodate:

I honestly think if four people came and said, I wanna do this program, that [the manager] probably would say, ok, everybody that smokes, we got four interested people wantin’ to take this program…But it’s been so varied when people try. Somebody wants to try gum, somebody wants to try a patch, somebody wants to go to this or that…It’s never been where a group has come in and said, this is what we wanna do.

### Facilitators or support needed

Based on participants’ descriptions of the facilitators to or support needed for anti-smoking initiative adoption and implementation, the following themes arose: high initiative compliance; Iowa Smokefree Air Act as facilitator; strong support system; resources for adoption.

#### Smoking policies

Eleven participants (35%) described having no issues with employee compliance as a facilitator to policy implementation. Eight participants (26%) also discussed the Iowa Smokefree Air Act as a facilitator (e.g., “the state has suggested that that’s how they want it done…the state law probably made it simpler to implement”). Some of the participants who already had a smoking policy described the Iowa Smoke Free Air Act as providing them with an opportunity to adopt a stronger policy (e.g., “I think the Smokefree Air Act gave us an opportunity to limit it more”). One of the facilitators unique to participants working for health-related agencies was the strong support they received from others (e.g., “We have a very supportive system of that here at public health…We’re very fortunate to have that mindful awareness of the health risks and be open to supporting change in those avenues”).

#### Smoking cessation activities

Among the 21 participants whose workplace offered activities, five (24%) indicated that no specific facilitators to adoption or implementation existed. Another five participants (24%) discussed support received as a facilitator. Some of this support was internal, such as having supportive management or staff (e.g., “We have a director that’s willing to be very progressive…to be able to help us figure out ways to be able to offer [activities], even with the lack of funding”). Other participants described external support, such as help from an employee assistance program. Three participants (14%) working for health-related agencies described easy access to cessation materials as one facilitator (e.g., “we have a lot of stuff that comes free through the state”).

Among the 11 participants whose workplace did not offer cessation activities, three (27%) noted that nothing in particular would help or support their workplace in offering them. Four (36%) participants indicated that financial resources or other forms of support would hypothetically facilitate the adoption process. Three participants (27%) described turning to external resources or organizations to decide what activities to adopt and implement if needed (e.g., “…we would no doubt turn to area health care providers and/or our insurance provider to help us create a program”).

### Small vs. very small workplaces

#### Smoking policies

Table 2 provides a summary of policy differences by workplace size. While participants coming from both small and very small workplaces described similar restrictions on smoking at their workplace, a greater proportion of participants from small workplaces described having a written smoking policy (87% vs. 63%), restricting outdoor smoking (80% vs. 83%), and restricting other forms of tobacco (47% vs. 25%). Fewer participants at very small workplaces described issues with adherence (13% vs. 27%). The most common reason for having a smoking policy among both small and very small workplaces was external smoking laws (e.g., Iowa Smokefree Air Act). Small workplaces also frequently cited wanting to create a healthy workplace.

**Table 2:** Smoking policies: small vs. very small workplaces

	Small workplaces (*n* = 15)	Very small workplaces (*n* = 17)
Has smoking policy	15	16
Has written smoking policy	13	10
Indoor smoking prohibited	15	16
Outdoor smoking restrictions	12	10
Smoking restricted in specific contexts	4	4
Other forms of tobacco restricted	7	4
Policy in place prior to Iowa Smokefree Air Act	8	6
Had issues with employee adherence to policy	4	2
Reasons for having a smoking policy		
■ External smoking laws	7	5
■ Healthy employees and workplace	5	1
■ Reduced organizational costs	2	1
■ Smoking is inappropriate, workplace image	3	3
■ Specific experience or incident	2	1
Challenges to adoption and implementation		
■ No challenges	9	6
■ Issues with adherence	3	4
■ How to enforce policy	1	1
Facilitators to adoption and implementation		
■ No issues with compliance	5	6
■ External smoking laws	3	5
■ Support from others (health agencies)	2	1

#### Smoking cessation activities

Table 3 provides a summary of activity differences by workplace size (note: since only one participant coming from a small workplace indicated that their company did not offer cessation activities, comparisons by workplace size for workplaces not offering activities were not made). A smaller proportion of very small workplaces offered cessation activities (41% vs. 93%, respectively). Participants coming from very small workplaces more commonly described offering information and referrals to services, compared to other activities. Both groups described similar reasons for adoption, as well as similar challenges and facilitators. Participants coming from small workplaces, however, more commonly described cost as a potential challenge (27% vs. 6%) and support from others as a facilitator (27% vs. 6%) to adoption and implementation.

**Table 3:** Smoking cessation activities: small vs. very small workplaces

	Small workplaces (*n* = 15)	Very small workplaces (*n* = 17)
Offers smoking cessation activities	14	7
Types of activity offered		
■ Information and referral to other services	7	5
■ Insurance coverage for cessation medication	6	2
■ Tobacco services through EAP or contracted vendor	6	1
■ Smoking cessation materials (e.g., magnets)	2	1
■ Smoking cessation classes	2	1
■ Insurance discount for non-smokers	1	0
Activities offered for at least 5 years	8	7
Current employee participation in cessation activities	0	0
Reasons for offering activities		
■ Healthy employees	4	3
■ Organizational benefits	4	2
■ Desire to help employees quit smoking	1	2
Challenges to adoption and implementation		
■ No challenges	5	4
■ Cost, lack of resources	4	1
■ Lack of employee interest in activities	2	2
Facilitators to adoption and implementation		
■ No specific facilitators described	4	1
■ Support from others (e.g., management)	4	1
■ Easy access to materials (health agencies)	1	2

## Discussion

This study offers valuable information about the anti-smoking initiatives that exist within some smaller workplaces, an understudied group. Even though over 20 states have implemented smoke-free laws, this study is one of few to examine anti-smoking initiatives at smaller workplaces in the context of such laws. In this study, indoor smoking policies were extremely widespread. However, more comprehensive initiatives (e.g., includes outdoor restrictions, all forms of tobacco) were less common, especially among very small workplaces. Previous studies have described a lack of resources and expertise as two major barriers to adopting health promotion programming at smaller workplaces [8, 10]. While this certainly may be true, findings from this study indicate that workplace management at some very small workplaces may not believe that these initiatives, particularly smoking cessation activities, are even needed in the first place.

By virtue of being so small, fewer total smokers may exist at very small workplaces. In the current study, smoking rates were fairly low, with 41% of participants indicating that no smokers existed within their organization. Having such few smokers may also explain why participation in cessation activities was almost non-existent. However, several participants prefaced this information by indicating they were not certain how many smokers existed within their organization, and were only providing an estimate based on their current knowledge. In some cases, smokers may conceal their smoking during work, since management tends to be more visible within these organizations [10] and the social acceptability of smoking has declined significantly over the past 50 years [38].

Given these factors, management may fail to accurately perceive the needs and characteristics of their employees. In fact, previous qualitative research has found differences in endorsed activities between employers and current smoking employees [39]. Regardless of how many smokers exist within an organization, having these activities in place demonstrates an organizational commitment to wellness and ensures that current and future employees can utilize these services if needed. State prohibitions on smoking have helped some smaller workplaces to adopt smoking policies that they may not have adopted otherwise. To continue to advance the field of tobacco control, other policies described by participants of this study that could be required by state law include not allowing smoking while on the clock and restricting other forms of tobacco use such as e-cigarettes. To increase the number of very small workplaces that offer cessation activities, policy makers could implement laws that require employers to do so.

Given the limited number of resources that many smaller workplaces have [8–10], requiring employers at these workplaces to implement on-site or high-cost programs for cessation is likely too burdensome. Instead, employers could be required to refer employees who express an interest in cessation to off-site evidence-based programs (e.g., state quit line), a more feasible approach to increasing employee access to these activities. Other opportunities include networking or resource sharing among organizations. For example, having organizations that have successfully implemented anti-smoking initiatives work with organizations currently going through the implementation process in order to increase chances of success. Requiring or encouraging these types initiatives at the state or federal level could help to eliminate disparities in adoption. It could also increase employer motivation and sense of urgency to provide more comprehensive anti-smoking initiatives, regardless of how many smokers currently work for the organization.

### Future research

Future research should consider organizational characteristics important at smaller workplaces, which could be modified to increase the chances of initiative adoption and success. As mentioned earlier, previous researchers have suggested that smaller workplaces have a more “intimate” work culture [10]. This presents a unique opportunity to work with management, who may be instrumental in getting employees to engage in health promotion efforts, to help them understand and communicate the importance of having these initiatives at their workplace.

It is also important to be mindful of the environmental context at smaller workplaces. Too often researchers develop and test health promotion programs at larger workplaces, which may have fewer or different contextual challenges (e.g., rates of turnover, financial stability). Interventions should be adapted to fit the specific needs of smaller workplaces. For example, one study testing a worksite wellness program for small businesses (less than 500 employees) found that even the smallest workplaces were willing to participate when provided with tailored direction and access to program resources [40]. This support may be crucial for very small workplaces, where management may not perceive a strong need for anti-smoking initiatives but willing to adopt them if given the right opportunities.

### Strengths and limitations

By interviewing key informants from smaller workplaces, this study gained more in-depth information about the context for tobacco control at these workplaces. Since key informants primarily held upper management or administrative positions, most participants provided a strong assessment of their workplace’s tobacco control efforts. Regarding limitations, recruitment of key informants occurred through the Business Leadership Network, where members have already expressed an interest in public health through participation in Network activities (e.g., attending public health issue forums). Study participants may have been more willing to discuss issues related to health compared to others, or their workplace more likely to have comprehensive initiatives. Nevertheless, results are likely to be relevant to similar settings and populations. These results may be particularly relevant for states similar to Iowa, where over a third of the population resides in rural areas [41], which are shown to have fewer voluntary restrictions related to smoking [42].

Several participants in this study came from organizations providing health-related services and could differ from organizations in other industries. For example, health-related agencies may be more likely to place greater restrictions on smoking. However, when comparing the responses between participants from these and other workplaces many of the same themes arose. This suggests that the findings from health-related agencies may sometimes apply to certain workplaces with other industries as well. Quantitative assessment of the relationship between industry and anti-smoking initiative adoption among a larger sample of smaller workplaces would provide additional insight into potential differences and similarities in tobacco control context.

## Conclusions

The very small workplaces in this study were less likely to have comprehensive anti-smoking initiatives. State laws restricting smoking have helped some smaller workplaces to adopt a smoking policy. Despite the widespread nature of these laws, differences in initiative adoption between small and very small workplaces in the current study existed. State laws placing additional restrictions on tobacco use in the workplace and requirements for offering cessation activities can help to reduce these disparities. Public health practitioners should work with policy makers to develop and implement these restrictions. Practitioners should also work with management to communicate the importance of having smoking policies and programs in place. Future research using quantitative study designs (e.g., environmental scans) can provide more generalizable insight into the comprehensiveness of anti-smoking initiatives within smaller workplaces. In doing so, more tailored efforts to reduce smoking behavior and improve employee health can be developed.
